# Advancing the frontiers of ovarian cancer therapy: a comprehensive synthesis of emerging cell death paradigms

**DOI:** 10.3389/or.2026.1850292

**Published:** 2026-07-28

**Authors:** Xi Ye, Weiping Zhu, Xiaojie Sun, Fangqiumei Yu, Qinhong Shen, Yingao Zheng, Xuanxuan Hong, Le Yu, Hanwen Zhang, Xiangfeng Zhang, Liehong Wang

**Affiliations:** 1 Qinghai University Affiliated Clinical Medical College, Xining, Qinghai, China; 2 Hefei Maternal and Child Health Hospital, Hefei, Anhui, China; 3 Hebei Children’s Hospital, Shijiazhuang, Hebei, China; 4 Qingdao Ruiside Hospital, Qingdao, Shandong, China; 5 Shanghai Hospital of Traditional Chinese Medicine, Shanghai, China; 6 Anhui Provincial Second People’s Hospital, Hefei, Anhui, China; 7 Hefei Eighth People’s Hospital, Hefei, Anhui, China; 8 Qinghai Red Cross Hospital, Xining, Qinghai, China

**Keywords:** cell death, development mechanism, ovarian cancer, regulatory mechanism, therapeutic target

## Abstract

Ovarian cancer is one of the most lethal malignancies of the female reproductive system, largely because many patients are diagnosed at an advanced stage and eventually develop recurrent or treatment-resistant disease. Although cytoreductive surgery, platinum-based chemotherapy, PARP inhibitors, anti-angiogenic agents, and immunotherapy have improved management for selected patients, relapse and drug resistance remain major clinical barriers. Regulated cell death pathways, including apoptosis, necroptosis, pyroptosis, ferroptosis, cuproptosis, disulfidptosis, and autophagy-dependent cell death, are increasingly recognized as contributors to ovarian cancer progression, immune regulation, and therapeutic response. This review summarizes current evidence on these pathways in ovarian cancer, with emphasis on key regulators, mechanistic links to treatment resistance, potential biomarkers, and the limitations that currently restrict clinical translation.

## 1 Introduction

Ovarian cancer remains a major cause of cancer-related mortality among women worldwide (1). Early-stage disease is often asymptomatic and effective population-level screening remains limited (2). As a result, many patients are diagnosed with stage III or IV disease, when tumor dissemination, recurrence risk, and treatment resistance are more pronounced (3). Standard management now includes cytoreductive surgery, platinum-taxane chemotherapy, PARP inhibitor-based maintenance therapy, anti-angiogenic treatment, and, in selected contexts, immunomodulatory approaches; however, durable disease control is still difficult to achieve in a substantial proportion of patients (4–6). These limitations have increased interest in biological processes that determine whether ovarian cancer cells survive therapeutic stress or undergo cell death.

Cell death pathways are central to tissue homeostasis, immune surveillance, and tumor suppression. The International Committee on the Nomenclature of Cell Death distinguishes accidental cell death (ACD), which results from overwhelming physical or chemical injury, from regulated cell death (RCD), which is mediated by defined molecular programs (7,8). In cancer, RCD is particularly relevant because tumor cells often acquire mechanisms to evade death, whereas therapeutic agents may act by restoring or redirecting death signaling. Major RCD modalities discussed in this review include apoptosis, necroptosis, pyroptosis, ferroptosis, cuproptosis, disulfidptosis, and autophagy-dependent cell death (9,10). Rather than providing an encyclopedic account of each pathway, this review focuses on their ovarian cancer-related evidence, translational relevance, and current uncertainties.

### 1.1 Literature search strategy

This review was designed as a narrative review informed by a structured literature search. A PubMed search was conducted to identify studies published between January 2021 and January 2026 that addressed regulated cell death pathways in ovarian cancer. The search strategy combined terms related to ovarian cancer with terms related to cell death, including “ovarian cancer,” “ovarian carcinoma,” “cell death,” “regulated cell death,” “apoptosis,” “necroptosis,” “pyroptosis,” “ferroptosis,” “cuproptosis,” “disulfidptosis,” and “autophagy-dependent cell death.” The Boolean search framework was as follows: (“ovarian cancer” OR “ovarian carcinoma”) AND (“cell death” OR “regulated cell death” OR apoptosis OR necroptosis OR pyroptosis OR ferroptosis OR cuproptosis OR disulfidptosis OR “autophagy-dependent cell death”).

Original studies, reviews, and mechanistic or translational studies relevant to ovarian cancer biology, therapeutic resistance, tumor microenvironment regulation, biomarkers, or treatment strategies were considered eligible. Studies focusing exclusively on non-ovarian malignancies, unrelated gynecological diseases, non-regulated forms of cell injury, or articles without accessible English abstracts were excluded. Given the narrative and integrative purpose of this review, the included literature was not subjected to formal meta-analysis. Instead, the evidence was synthesized qualitatively, with attention to whether findings were derived from cell-line experiments, animal models, bioinformatic analyses, retrospective clinical cohorts, or prospective clinical studies.

Given the narrative and integrative purpose of this review, the literature selection process was described qualitatively rather than presented as a formal PRISMA flow diagram. The search strategy was used to guide literature identification and thematic synthesis, while the final inclusion of studies was based on relevance to ovarian cancer biology, regulated cell death mechanisms, therapeutic resistance, tumor microenvironment regulation, biomarkers, and translational implications.

### 1.2 Evidence appraisal and synthesis

Because the evidence supporting different cell death modalities in ovarian cancer is heterogeneous, the included literature was appraised according to the type and maturity of evidence. Evidence was broadly categorized as: (1) mechanistic preclinical evidence from ovarian cancer cell lines or animal models; (2) bioinformatic or transcriptomic evidence derived from public datasets or patient-derived molecular profiles; (3) retrospective clinical or pathological association studies; and (4) prospective clinical evidence or interventional trial data. When interpreting therapeutic relevance, greater weight was given to findings supported by ovarian cancer-specific experimental validation or clinical cohorts, whereas associations based solely on computational analyses or extrapolation from other tumor types were described as exploratory.

To improve readability and transparency, the principal evidence for each cell death modality is summarized in Table 1, including key regulators, ovarian cancer-specific evidence, proposed biomarkers or therapeutic agents, and the current stage of translational maturity. This approach was used to distinguish relatively mature areas, such as apoptosis- and ferroptosis-related therapeutic resistance, from emerging areas, such as cuproptosis, disulfidptosis, and autophagy-dependent cell death, where direct ovarian cancer-specific evidence remains more limited. Accordingly, composite indices and multi-parametric death-related signatures discussed in this review, including the Cell Death Index (CDI) and Death Vulnerability Signature (DVS), should be interpreted as conceptual or hypothesis-generating frameworks unless supported by independent clinical validation. Bioinformatic associations, prognostic correlations, and pathway enrichment results can generate biologically plausible hypotheses, but they do not by themselves establish functional causality or immediate clinical utility. Before such frameworks can guide clinical decision-making, they require standardized molecular panels, reproducible assay platforms, predefined cut-off values, independent validation cohorts, and prospective evaluation in clinically annotated ovarian cancer populations.

**TABLE 1:** Evidence maturity of major cell death modalities in ovarian cancer.

Cell death modality	Key regulators	Main ovarian cancer-specific evidence	Proposed biomarkers/therapeutic agents	Evidence type	Translational maturity
Apoptosis	BCL-2 family proteins, caspase-8/9/3, p53, FAS/FASL, TRAIL receptors	Apoptotic dysregulation is closely linked to platinum resistance, survival signaling, and response to cytotoxic chemotherapy in ovarian cancer	BCL-2/BCL-XL inhibitors, TRAIL pathway modulators, platinum agents, PARP inhibitor combinations	Cell-line studies, animal models, retrospective clinical correlations, selected clinical trial evidence	Relatively mature; clinically relevant but still context-dependent
Ferroptosis	GPX4, SLC7A11, ACSL4, FTH1, TFR1, NRF2	Ferroptosis has been associated with oxidative stress, lipid peroxidation, chemoresistance, and potential vulnerability to metabolic or redox-targeted therapy in ovarian cancer	GPX4 inhibitors, system Xc− inhibitors, iron metabolism modulators, sorafenib-like agents, ferroptosis-related gene signatures	Preclinical studies, bioinformatic analyses, limited clinical correlations	Active translational area; most therapeutic evidence remains preclinical
Pyroptosis	NLRP3, AIM2, caspase-1/4/5, gasdermin D/E, IL-1β, IL-18	Pyroptosis may influence inflammatory signaling, immune activation, tumor microenvironment remodeling, and prognosis-related molecular signatures in ovarian cancer	Gasdermin-related markers, inflammasome components, pyroptosis-related gene scores	Bioinformatic analyses, mechanistic studies, limited clinical association studies	Emerging; functional validation remains insufficient
Necroptosis	RIPK1, RIPK3, MLKL, TNFR1, FADD, caspase-8	Necroptosis is implicated in inflammatory tumor cell death, immune microenvironment regulation, prognosis, and potential sensitization to therapy	RIPK1/RIPK3/MLKL-related signatures, necroptosis-associated lncRNAs, necroptosis-inducing agents	Preclinical studies, transcriptomic analyses, retrospective prognostic studies	Emerging to intermediate; clinical utility not yet established
Cuproptosis	FDX1, DLAT, LIAS, LIPT1, ATP7A/ATP7B, copper transport regulators	Cuproptosis-related genes have been linked to metabolic vulnerability, mitochondrial function, prognosis, and immune infiltration in ovarian cancer, but direct functional evidence remains limited	Cuproptosis-related gene signatures, copper ionophores, copper transport regulators	Mainly bioinformatic analyses and mechanistic extrapolation; limited ovarian cancer-specific validation	Early exploratory stage
Disulfidptosis	SLC7A11, G6PD, NADPH-related pathways, actin cytoskeleton regulators	Disulfidptosis may be relevant to redox imbalance and cytoskeletal collapse in metabolically stressed tumor cells, but ovarian cancer-specific evidence remains sparse	SLC7A11-related signatures, redox metabolism markers	Primarily computational or extrapolative evidence	Early exploratory stage
Autophagy-dependent cell death	ATG proteins, Beclin-1, LC3, ULK1, VPS34, mTOR	Autophagy has dual roles in ovarian cancer, contributing to stress adaptation, chemotherapy resistance, and, under specific conditions, autophagy-dependent cell death	Autophagy modulators, mTOR pathway inhibitors, LC3/Beclin-1-related markers	Preclinical studies, clinical correlations, limited interventional evidence	Intermediate; therapeutic direction remains context-dependent
Necrosis	Hypoxia-related pathways, ATP depletion, ROS, DAMPs, HMGB1	Tumor necrosis reflects hypoxia, metabolic stress, and aggressive tumor biology, and may influence inflammation and immune remodeling in ovarian cancer	Hypoxia markers, HMGB1, DAMP-associated inflammatory markers	Pathological observations, mechanistic inference, clinical associations	Clinically observable but not a precise therapeutic target

## 2 Cell death

### 2.1 Accidental cell death

**FIGURE 1:**
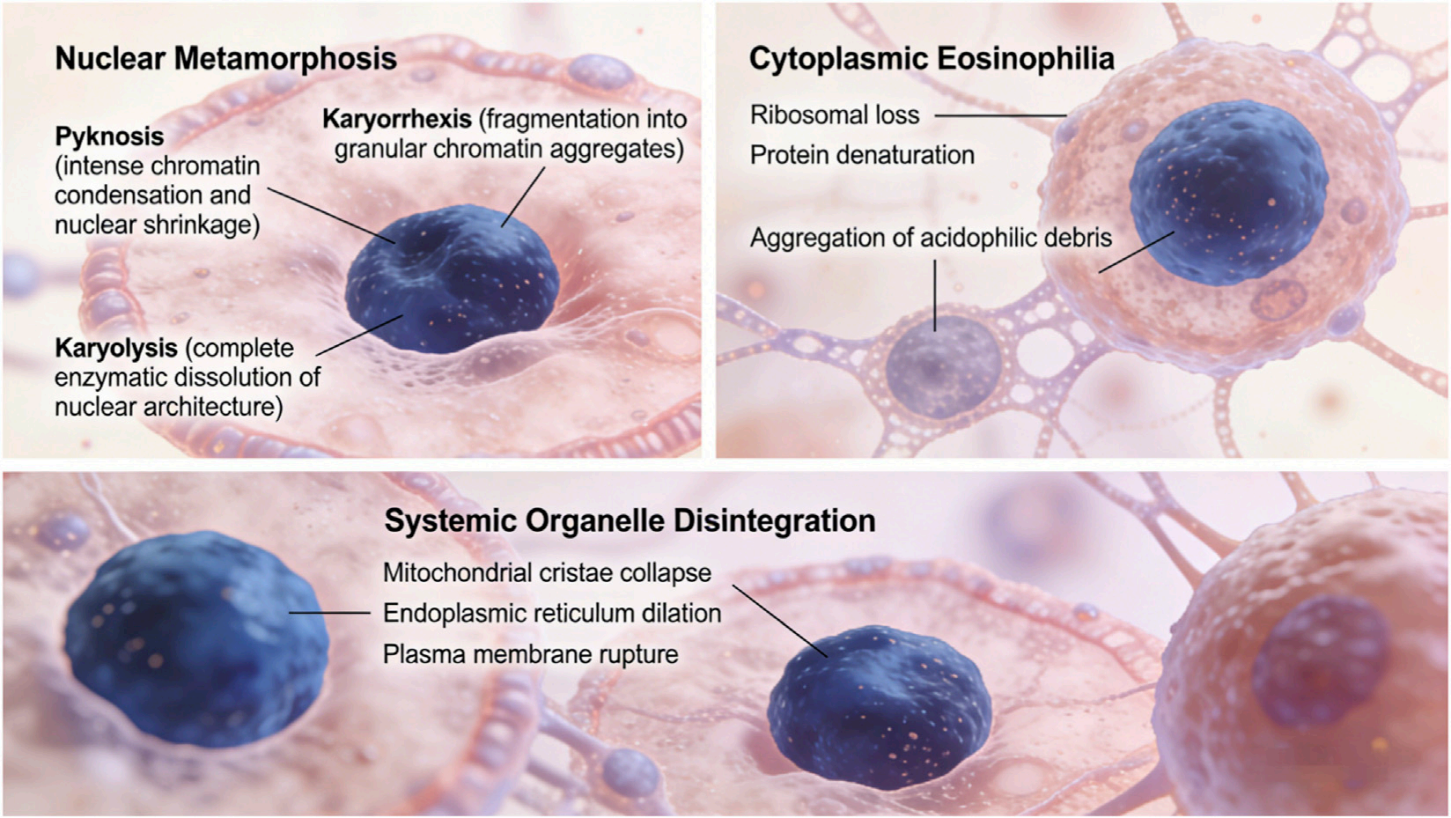
Schematic overview of necrosis. Necrosis is characterized by severe disruption of cellular homeostasis, leading to ion imbalance, cellular swelling, lysosomal damage, organelle breakdown, and plasma membrane rupture. The loss of membrane integrity results in the release of intracellular contents, including DAMPs, nucleic acids, HMGB1, ATP, and inflammatory mediators, which can activate local inflammation and amplify tissue injury.

Accidental cell death, commonly referred to as necrosis, occurs when severe external or internal stress disrupts cellular homeostasis beyond recovery. Typical triggers include extreme pH changes, radiation, infection, immune-mediated injury, hypoxia, and other insults that impair ion gradients and membrane integrity. Necrotic cells show swelling, organelle disruption, lysosomal damage, and plasma membrane rupture, followed by release of intracellular contents such as damage-associated molecular patterns (DAMPs), nucleic acids, and inflammatory mediators (11). Histologically, necrosis is characterized by nuclear pyknosis, karyorrhexis, or karyolysis, increased cytoplasmic eosinophilia, and organelle breakdown (see Figure 1).

Although necrosis has traditionally been regarded as unregulated injury, several biochemical events can shape its progression, including reactive oxygen species (ROS) accumulation, calcium overload, calpain activation, and poly-ADP-ribose polymerase (PARP)-mediated depletion of NAD+ and ATP (12). Unlike apoptosis, which is usually cleared without major membrane rupture, necrosis releases IL-1β, TNF-α, HMGB1, and other DAMPs, thereby promoting inflammation and potentially amplifying tissue injury (13).

### 2.2 Regulated cell death

#### 2.2.1 Necroptosis

Necroptosis is a regulated, lytic form of cell death that shares morphological features with necrosis but is controlled by specific signaling molecules. It can be initiated by TNFα/TNFR1, Fas/FasL, Toll-like receptors, and intracellular nucleic acid sensors such as RIG-I and STING. These receptors may promote survival and inflammation through NF-κB signaling under some conditions; however, when caspase-8 activity is inhibited or insufficient, signaling can shift toward necroptosis (14).

**FIGURE 2:**
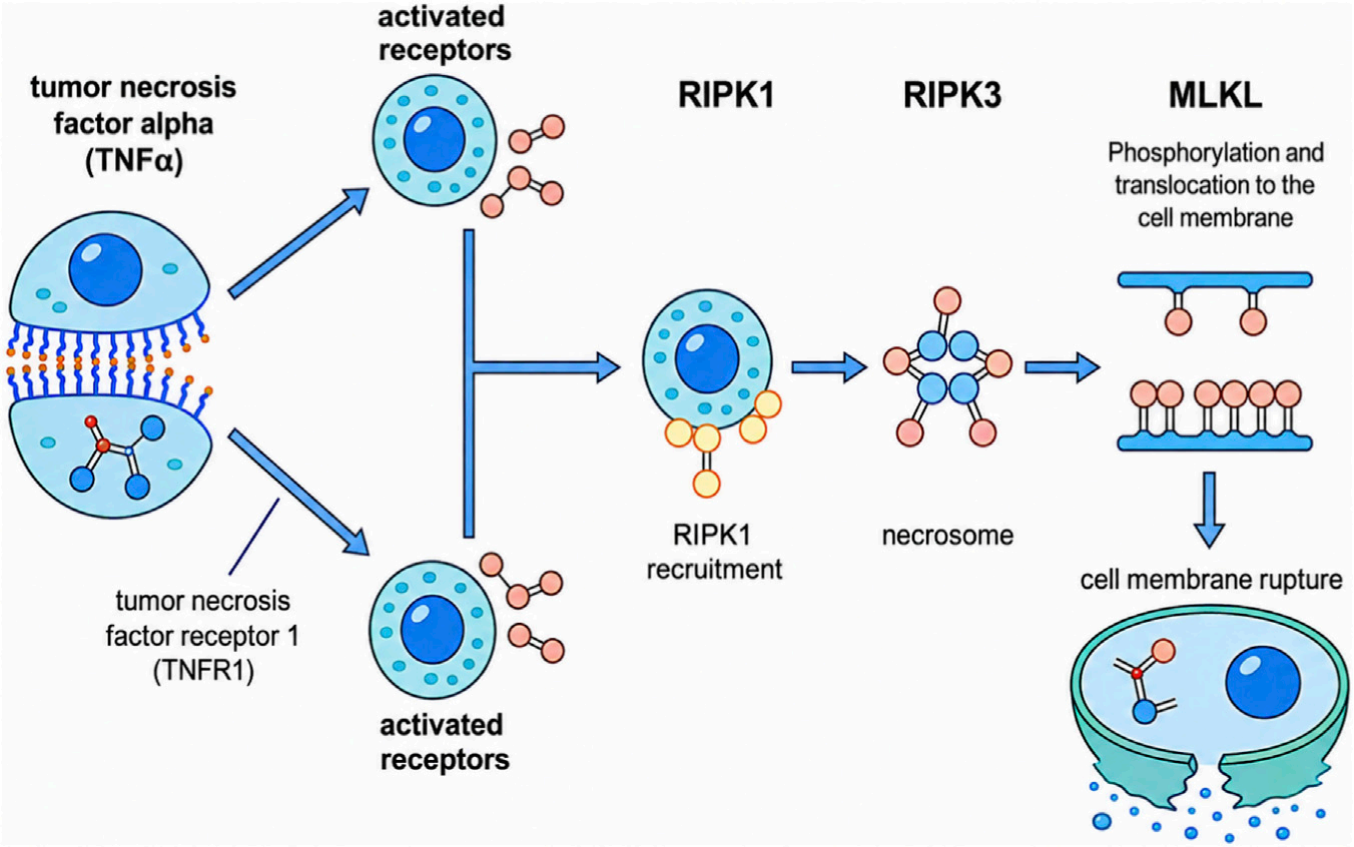
Canonical signaling pathway of necroptosis. Necroptosis is commonly initiated by death receptor or innate immune receptor signaling, including TNFα/TNFR1, Fas/FasL, Toll-like receptors, RIG-I, and STING. When caspase-8 activity is insufficient or inhibited, RIPK1 and RIPK3 form a necroptotic signaling complex, leading to MLKL phosphorylation, oligomerization, plasma membrane translocation, pore formation, and lytic cell death.

The canonical necroptosis pathway is mediated by RIPK1, RIPK3, and mixed-lineage kinase domain-like protein (MLKL). Deubiquitination of RIPK1 facilitates recruitment of RIPK3 and formation of a RIPK1-RIPK3 signaling complex. RIPK3 then phosphorylates MLKL, leading to MLKL oligomerization, membrane translocation, pore formation, and loss of plasma membrane integrity (15,16). Earlier models implicated mitochondrial PGAM5/DRP1 signaling, but subsequent genetic and pharmacological studies indicate that plasma membrane disruption is the principal terminal event in TNF-induced necroptosis (17) (Figure 2).

#### 2.2.2 Apoptosis

**FIGURE 3:**
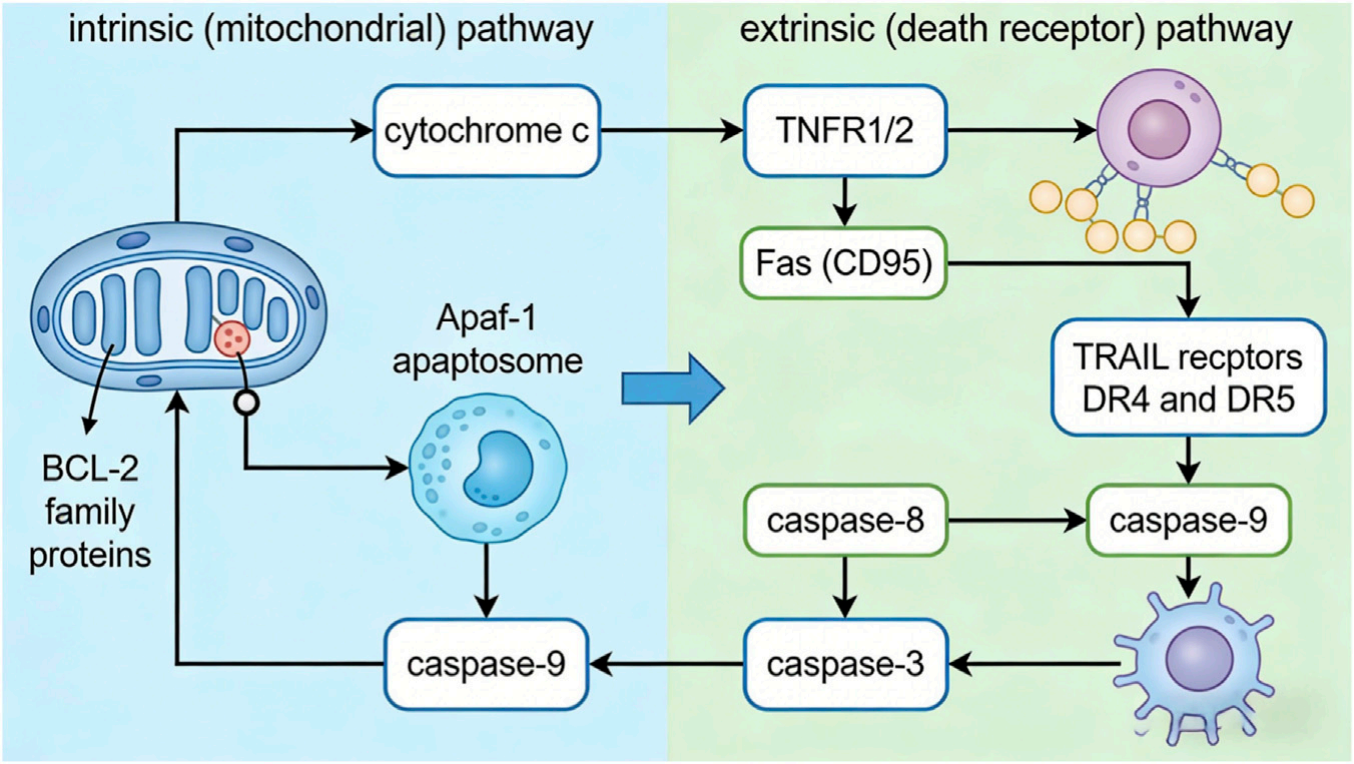
Intrinsic and extrinsic pathways of apoptosis. The intrinsic pathway is triggered by intracellular stress and is regulated by BCL-2 family proteins, which control mitochondrial outer membrane permeabilization, cytochrome c release, apoptosome formation, caspase-9 activation, and downstream caspase-3/7 execution. The extrinsic pathway is initiated by death ligands such as TNFα, FasL, or TRAIL binding to their receptors, resulting in DISC formation, caspase-8 activation, and direct or mitochondria-amplified executioner caspase activation.

Apoptosis is a non-lytic form of programmed cell death mediated mainly through intrinsic mitochondrial and extrinsic death receptor pathways (18) (Figure 3). In the intrinsic pathway, genotoxic stress, oxidative injury, or cytotoxic therapy promotes mitochondrial outer membrane permeabilization (MOMP). This process is regulated by the BCL-2 family, including anti-apoptotic proteins such as BCL-2 and BCL-XL, pro-apoptotic effectors such as BAX and BAK, and BH3-only regulators such as BIM, BID, PUMA, BAD, and NOXA. MOMP enables cytochrome c release, apoptosome formation, caspase-9 activation, and downstream caspase-3/7-mediated cellular dismantling.

The extrinsic pathway begins at the cell surface after binding of death ligands such as TNFα, FasL, or TRAIL to their receptors. Receptor trimerization promotes assembly of death-inducing signaling complexes containing adaptor proteins such as TRADD and FADD and initiator caspases such as caspase-8 or caspase-10. Activated caspase-8 can directly activate executioner caspases or cleave BID to tBID, thereby linking death receptor signaling to mitochondrial apoptosis. Apoptotic cells typically show chromatin condensation, nuclear fragmentation, membrane blebbing, and formation of apoptotic bodies that can be removed by phagocytes (19).

#### 2.2.3 Pyroptosis

**FIGURE 4:**
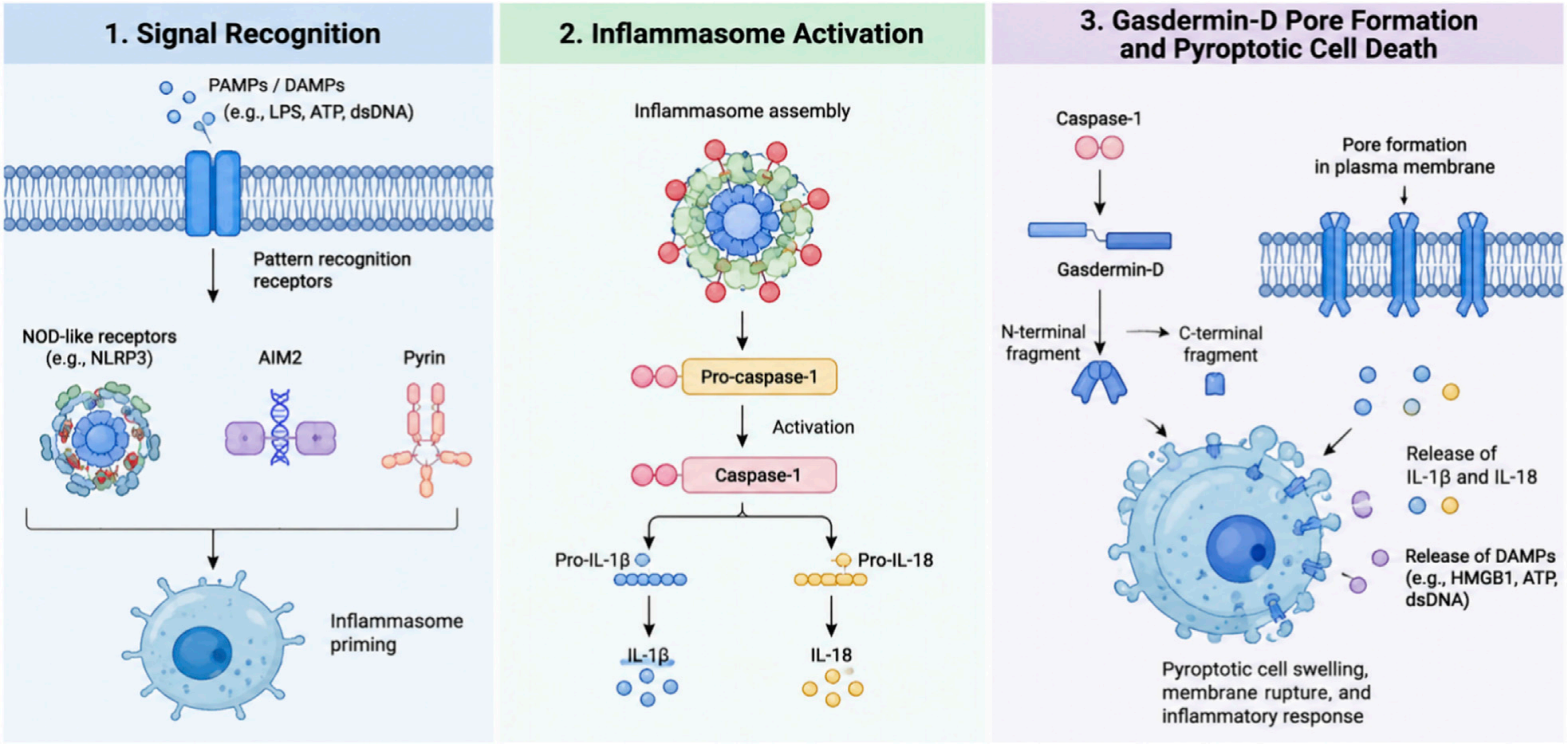
Inflammasome-mediated pyroptosis. Pyroptosis proceeds through signal recognition, inflammasome activation, and gasdermin-D-mediated pore formation. Pattern recognition receptors, including NOD-like receptors, AIM2, and Pyrin, detect PAMPs or DAMPs and promote inflammasome assembly. Activated caspase-1 cleaves pro-IL-1β and pro-IL-18 and also cleaves gasdermin-D, generating an N-terminal fragment that forms pores in the plasma membrane. This process leads to cell swelling, membrane rupture, and release of IL-1β, IL-18, DAMPs, and other inflammatory mediators.

Pyroptosis is an inflammatory, lytic form of regulated cell death mediated by inflammasome activation and gasdermin-dependent membrane pore formation. Inflammasome sensors, including members of the NOD-like receptor family, AIM2, and Pyrin, detect pathogen-associated or damage-associated signals and activate inflammatory caspases (20) (Figure 4). Cleavage of gasdermin D creates membrane pores, leading to cell swelling, membrane rupture, and release of IL-1β and IL-18. This response contributes to host defense but, when excessive or chronic, can drive pathological inflammation (21). NLRP3 is among the most widely studied inflammasome sensors and has been implicated in multiple inflammatory and metabolic disorders (22).

#### 2.2.4 Ferroptosis

**FIGURE 5:**
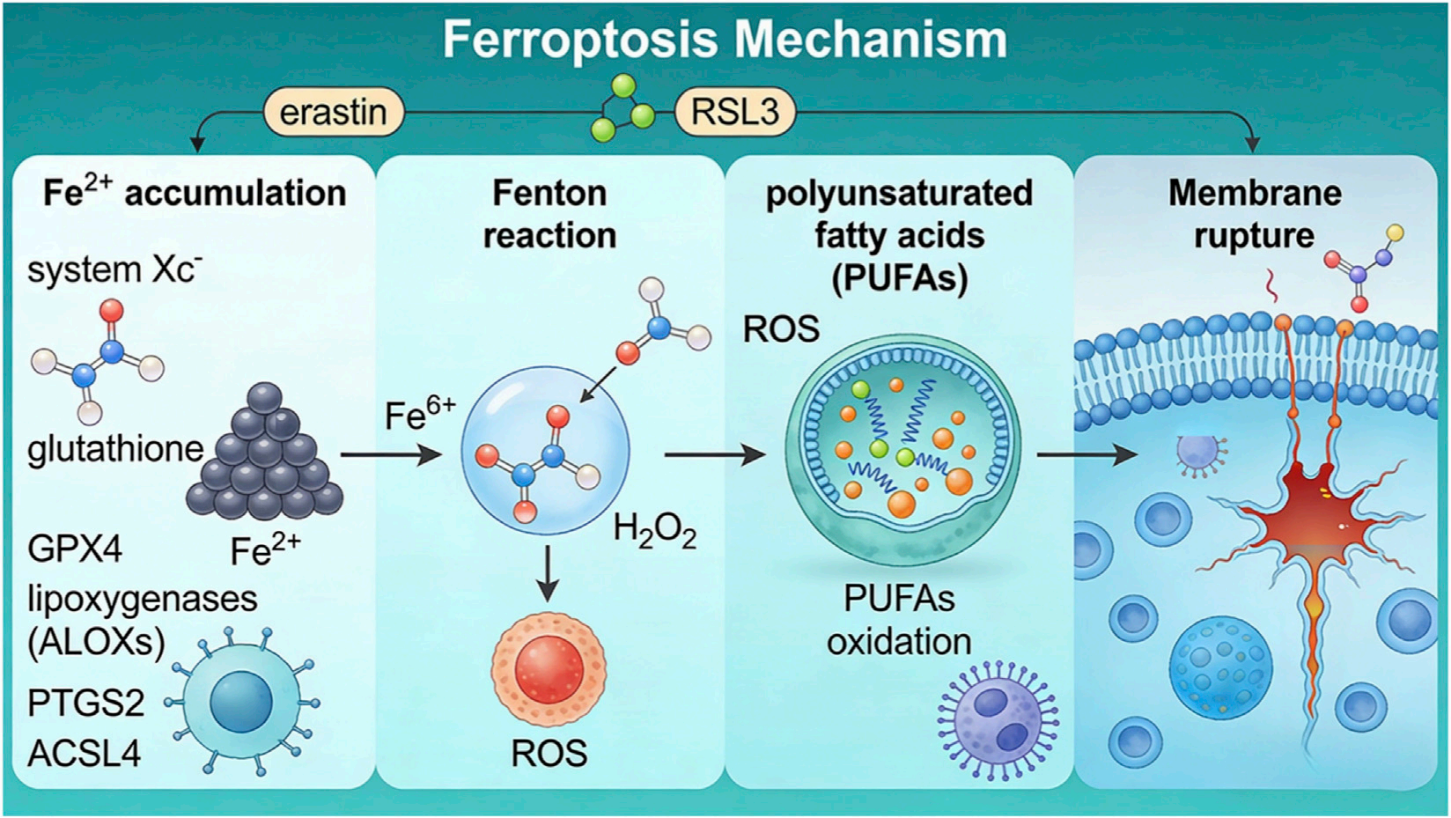
Core regulatory network of ferroptosis. Ferroptosis is driven by iron-dependent lipid peroxidation and failure of antioxidant defense systems. Labile iron promotes ROS generation and lipid peroxide accumulation, whereas the system Xc−–GSH–GPX4 axis limits lipid hydroperoxide toxicity. Key regulators such as SLC7A11, GPX4, ACSL4, PTGS2, FSP1, and iron metabolism-related proteins determine ferroptotic susceptibility and may influence treatment response in ovarian cancer.

Ferroptosis is an iron-dependent, oxidative form of regulated cell death characterized by accumulation of lipid peroxides in cellular membranes. Labile iron can promote lipid peroxidation through Fenton chemistry and through iron-dependent enzymes such as lipoxygenases. Classical ferroptosis inducers act through convergent mechanisms: erastin inhibits system Xc- and depletes glutathione, whereas RSL3 directly inhibits GPX4, a key enzyme that reduces lipid hydroperoxides (23) (Figure 5). Ferroptotic sensitivity is also influenced by enzymes that determine membrane lipid composition, including ACSL4, and by stress markers such as PTGS2 (24).

#### 2.2.5 Cuproptosis

**FIGURE 6:**
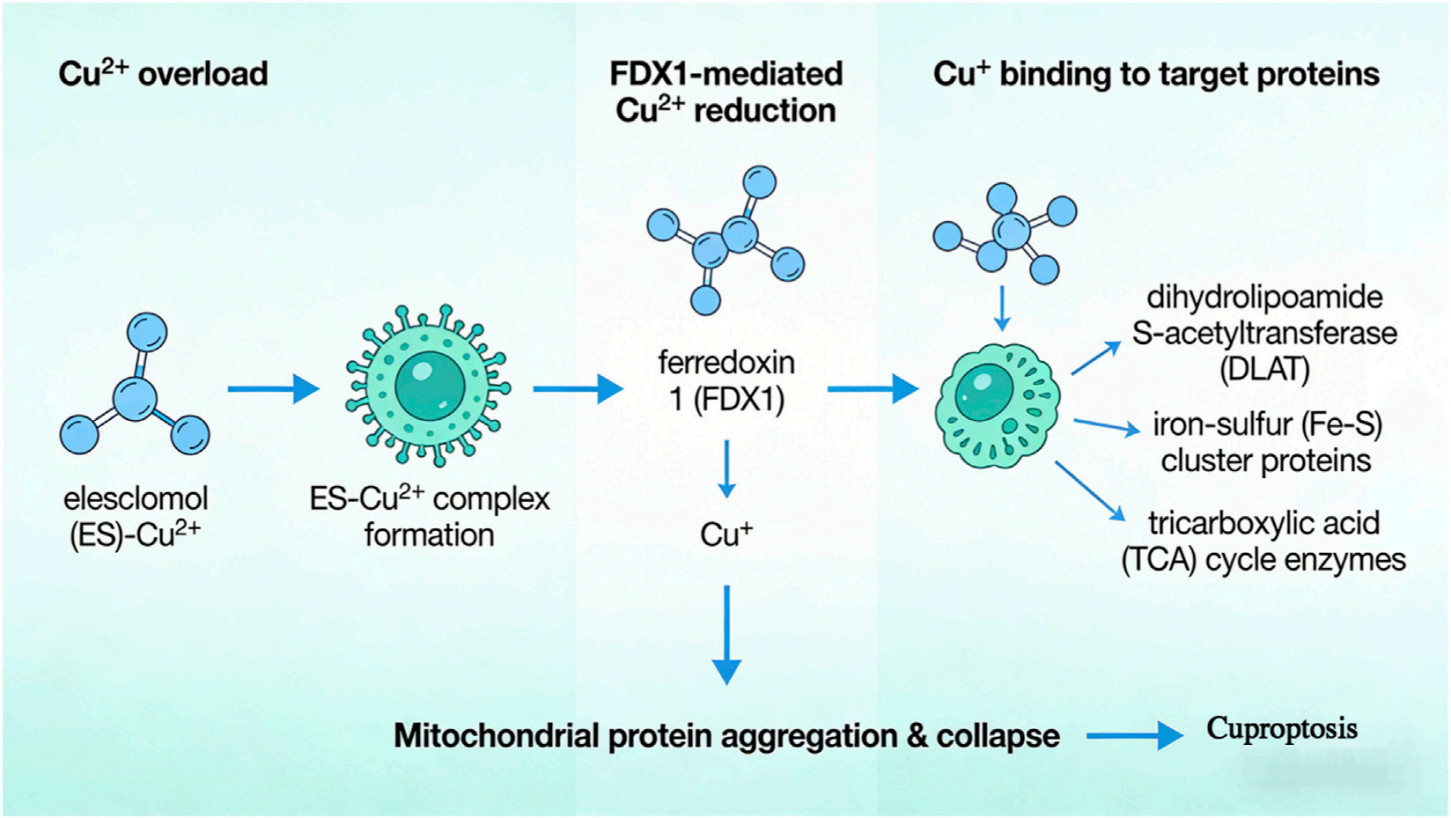
Molecular mechanism of cuproptosis. Cuproptosis is a copper-dependent form of regulated cell death associated with mitochondrial proteotoxic stress. Copper accumulation promotes binding to lipoylated tricarboxylic acid cycle proteins, especially DLAT, leading to protein aggregation, destabilization of iron–sulfur cluster proteins, impaired mitochondrial respiration, and cell death. FDX1 contributes to this pathway by regulating copper reduction and protein lipoylation.

Cuproptosis is a recently described form of regulated cell death driven by copper-dependent mitochondrial proteotoxic stress. Its core features include aggregation of lipoylated tricarboxylic acid cycle proteins, particularly dihydrolipoamide S-acetyltransferase (DLAT), and destabilization of iron-sulfur cluster proteins (25). Ferredoxin 1 (FDX1) contributes to this process by regulating copper reduction and protein lipoylation. When intracellular copper accumulates in cells with active mitochondrial metabolism, copper binding to lipoylated proteins can impair mitochondrial function and trigger cell death (26) (Figure 6).

#### 2.2.6 Disulfidptosis

**FIGURE 7:**
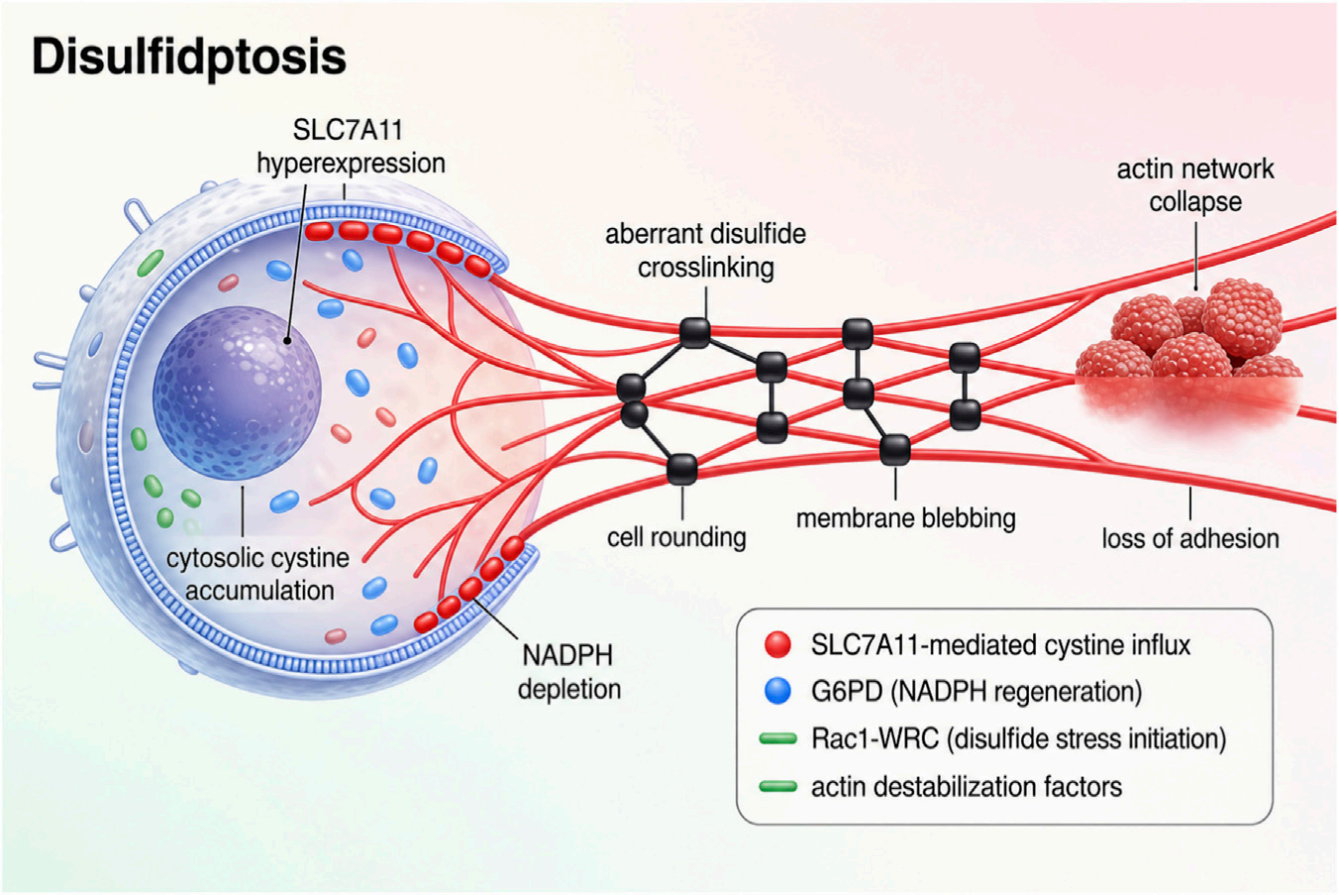
Proposed mechanism of disulfidptosis. Disulfidptosis is associated with disulfide stress under conditions of glucose deprivation and high SLC7A11 activity. SLC7A11-mediated cystine influx, NADPH depletion, and impaired thiol reduction promote abnormal disulfide crosslinking in actin and cytoskeleton-associated proteins. These changes cause cell rounding, cytoskeletal contraction, actin network collapse, loss of adhesion, and subsequent cell death.

Disulfidptosis is a recently proposed form of regulated cell death associated with disulfide stress and cytoskeletal collapse. It occurs particularly under glucose starvation in cells with high SLC7A11 activity. Increased cystine uptake and reduced NADPH availability impair thiol-redox balance, allowing excessive disulfide bond formation in actin and related cytoskeletal proteins (27,28). The resulting actin network contraction, cell rounding, and loss of adhesion differ from classical apoptosis or necrosis (29). Proposed regulators include SLC7A11, G6PD, and the Rac1-WAVE regulatory complex, although cancer-specific validation remains at an early stage (30) (Figure 7).

#### 2.2.7 Autophagy-dependent cell death

**FIGURE 8:**
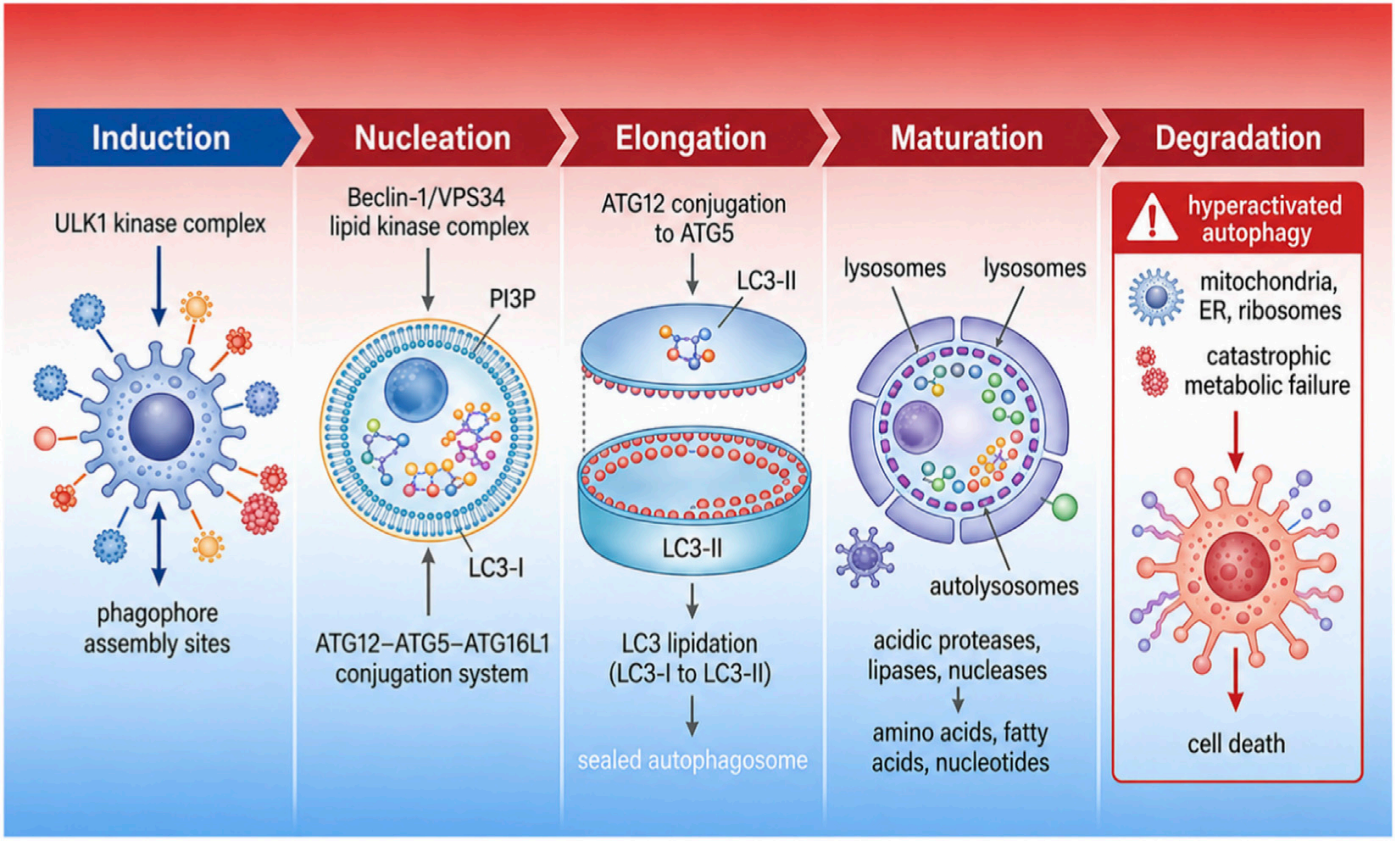
Autophagy and autophagy-dependent cell death. Autophagy begins with activation of upstream regulators such as the ULK1 complex, followed by phagophore nucleation through the Beclin-1/VPS34 complex, membrane elongation through ATG conjugation systems, LC3 lipidation, autophagosome formation, fusion with lysosomes, and cargo degradation. Although autophagy usually supports stress adaptation and survival, excessive or dysregulated autophagic flux may contribute to autophagy-dependent cell death under specific conditions.

Autophagy is a conserved catabolic process that maintains cellular homeostasis by delivering cytoplasmic components to lysosomes for degradation. In most contexts it supports survival under stress, but excessive or dysregulated autophagic flux can contribute to autophagy-dependent cell death (ADCD) (31,32). Autophagy includes macroautophagy, microautophagy, and chaperone-mediated autophagy; macroautophagy is the best characterized form and involves formation of double-membrane autophagosomes (33,34). The process is regulated by autophagy-related genes and proceeds through induction, nucleation, elongation, maturation, and lysosomal degradation (35–40). Key molecular components include the ULK1 complex, Beclin-1/VPS34, ATG12-ATG5-ATG16L1, and LC3 lipidation (Figure 8).

## 3 The association between cell death and ovarian cancer

### 3.1 Necrosis and ovarian cancer

In ovarian cancer, necrosis is mainly associated with hypoxia, nutrient deprivation, acidosis, rapid tumor expansion, and treatment-related injury. These conditions impair ATP production, disrupt ion homeostasis, damage mitochondria and lysosomes, and ultimately cause membrane rupture. Necrotic areas can therefore reflect an imbalance between tumor growth and vascular supply, but they may also influence the tumor microenvironment by releasing DAMPs and inflammatory mediators.

Hypoxia, nutrient stress, and acidosis are common in advanced ovarian tumors and can promote necrotic cell death. Hypoxia reduces mitochondrial oxidative phosphorylation and ATP production, which compromises Na+/K + -ATPase and Ca2+-ATPase activity and favors osmotic swelling and membrane damage (41,42). HIF-1α activation further supports glycolytic metabolism, aberrant angiogenesis, and lactate accumulation, thereby reinforcing hypoxia and acidosis (43). Nutrient deprivation limits protein synthesis and organelle maintenance, whereas the Warburg phenotype increases extracellular acidification and may aggravate membrane and lysosomal injury (44,45). At the molecular level, ATP depletion, ROS accumulation, lysosomal permeabilization, and DAMP release form a self-amplifying injury process (46–49). In ovarian cancer, the biological impact of necrosis is therefore context dependent: it may enhance immunogenic signaling, but persistent necrosis can also support chronic inflammation, angiogenesis, and metastatic niche formation.

### 3.2 Necroptosis and ovarian cancer

Necroptosis in ovarian cancer is mediated mainly through the RIPK1-RIPK3-MLKL axis (50). Activation of this pathway can induce lytic tumor cell death and release DAMPs and inflammatory cytokines, which may stimulate dendritic cells and adaptive immune responses. Preclinical data suggest that pharmacological induction of necroptosis, such as by AEZS-126, can suppress ovarian cancer cell growth and improve chemosensitivity (51). Reduced RIPK3 expression has been associated with advanced disease and worse overall survival, while necroptosis-related genes such as RIPK1, MLKL, and TNFRSF1A have been reported to be differentially expressed in ovarian tumors (52,53). However, the effect of necroptosis is not uniformly tumor suppressive. Excessive RIPK1-related signaling may increase IL-6 and TNF-α production, activate NF-κB, and promote proliferation, epithelial-mesenchymal transition, or multidrug resistance (54). Impaired MLKL-mediated execution may also allow tumor cells to avoid lytic death and acquire more aggressive phenotypes (55). Necroptosis can additionally reshape the immune microenvironment by influencing dendritic cell maturation, CD8^+^ T-cell infiltration, checkpoint expression, B-cell metabolic stress, NK/T-cell transcriptional states, and macrophage polarization (56–58). These observations support a dual role for necroptosis that depends on pathway intensity, spatial context, and immune status. Necroptosis-associated lncRNA signatures have also been reported to predict prognosis and distinguish immune “cold” and “hot” tumor phenotypes in ovarian cancer, supporting a potential link between necroptosis-related transcriptional programs and immune stratification (53).

### 3.3 Apoptosis and ovarian cancer

Apoptosis evasion is a major contributor to ovarian cancer progression and treatment resistance. It may result from altered expression of BCL-2 family proteins, TP53 mutation, impaired caspase activation, dysregulated death receptor signaling, activation of pro-survival pathways, or non-coding RNA-mediated regulation. These changes reduce mitochondrial priming and enable tumor cells to survive platinum- or taxane-induced stress.

The BCL-2 family is central to intrinsic apoptosis in ovarian cancer. Increased expression of anti-apoptotic proteins such as BCL-2, BCL-XL, or MCL-1 and reduced activity of BAX or BAK can prevent MOMP, cytochrome c release, and caspase activation (59). Experimental suppression of BCL-XL or related anti-apoptotic proteins can restore mitochondrial apoptosis and improve sensitivity to chemotherapy in selected models, whereas persistent overexpression is linked to chemoresistance and recurrence (60). These findings support continued investigation of BH3 mimetics and rational combinations, while also requiring careful attention to tumor subtype, biomarker selection, and toxicity.

Upstream survival pathways also regulate apoptosis in ovarian cancer. PI3K/AKT/mTOR signaling is frequently activated and can promote anti-apoptotic protein expression, inhibit pro-apoptotic factors, and reduce caspase activation (61–63). Inhibition of this axis has been reported to enhance cisplatin- and paclitaxel-induced apoptosis in preclinical models (64). Stress-activated MAPK signaling, including JNK and p38, can increase expression of pro-apoptotic regulators and sensitize cells to chemotherapy, whereas pathway suppression may contribute to drug resistance (65). Non-coding RNAs add another layer of regulation: several miRNAs and lncRNAs modulate BCL-2 family proteins, caspases, death receptors, and PI3K/AKT signaling (66–70). Restoring apoptosis through BH3 mimetics, death receptor agonists, pathway inhibitors, or RNA-based strategies therefore remains a plausible therapeutic direction, but most supporting evidence is preclinical or biomarker-based rather than definitive clinical validation (71–73).

### 3.4 Pyroptosis and ovarian cancer

Pyroptosis is relevant to ovarian cancer because it links tumor cell death with inflammatory and immune signaling. Gasdermin-mediated membrane rupture releases tumor antigens, DAMPs, IL-1β, and IL-18, which can promote dendritic cell activation and T-cell priming (74). However, chronic inflammasome activation may also support immunosuppressive inflammation, angiogenesis, and tumor progression. Several studies have associated pyroptosis-related genes and lncRNAs with prognosis, immune infiltration, and potential response to immunotherapy in ovarian cancer (75–77). These findings are useful for biomarker development, but many are based on transcriptomic correlations and require functional validation in ovarian cancer models and clinical cohorts.

### 3.5 Ferroptosis and ovarian cancer

Ferroptosis has attracted particular interest in ovarian cancer because it connects iron metabolism, lipid peroxidation, oxidative stress, and therapy response. Iron accumulation has been reported in ovarian tumor contexts and may contribute to ROS production, genomic instability, and malignant transformation (78). Altered expression of iron transporters and storage proteins, including TFRC, transferrin-related proteins, ferritin, and ferroportin, may influence ferroptotic vulnerability and tumor behavior (79,80).

Several ferroptosis-regulating systems have been implicated in ovarian cancer. The system Xc--GSH-GPX4 axis limits lipid peroxide accumulation, and high SLC7A11 or GPX4 expression can protect tumor cells from ferroptotic death (81–83). ACSL4, LPCAT3, and lipid-remodeling enzymes influence the availability of peroxidation-prone phospholipids (84). NRF2-related antioxidant programs, FSP1-CoQ10 signaling, and AMPKα-SCD1-mediated lipid remodeling can further modify ferroptotic sensitivity (85–90). Preclinical studies suggest that ferroptosis induction may enhance the effects of platinum agents, PARP inhibitors, or other therapies, but translation remains limited by drug specificity, delivery, toxicity, and the need for predictive biomarkers (91–96). Overall, ferroptosis appears to be one of the better-supported non-apoptotic death pathways in ovarian cancer, although most mechanistic and therapeutic data remain preclinical. Recent work on ferroptosis in female reproductive system disorders further supports the relevance of ferroptosis-related redox imbalance, lipid peroxidation, and therapeutic vulnerability in gynecological disease contexts, including ovarian cancer (24).

### 3.6 Cuproptosis and ovarian cancer

Copper homeostasis may influence ovarian cancer biology through mitochondrial metabolism, oxidative stress, angiogenesis, and treatment response. Copper-binding proteins and copper transporters, including CTR1 and ATP7A/B, have been linked to tumor behavior and platinum sensitivity in some studies (97–100). Copper-related signaling may also interact with hypoxia, immune regulation, and metabolic adaptation (101–104). However, the degree to which these observations directly reflect cuproptosis, rather than broader copper biology, should be interpreted cautiously (105–107).

In the cuproptosis pathway, FDX1, DLD, DLAT, and PDHB are key components of the lipoic acid and mitochondrial metabolic machinery. Dysregulation of these genes has been reported in ovarian tumors and associated with stage, chemoresistance, or survival in transcriptomic analyses (108). YY1 may regulate expression of components of this pathway, while FDX1 contributes to copper reduction and protein lipoylation (109–112). Studies of LIAS and related lipoylation factors also suggest a possible link between mitochondrial metabolism, copper sensitivity, and prognosis (113,114). These findings support cuproptosis as a promising but still emerging field in ovarian cancer.

Accordingly, the lipoic acid-copper axis should be presented as an exploratory therapeutic vulnerability rather than an established treatment strategy. Potential approaches include modulating copper transport, targeting FDX1-related copper activation, or altering lipoylation status, but these concepts require validation in ovarian cancer-specific models and, eventually, biomarker-driven clinical studies.

Cuproptosis-related gene profiles have also been used to classify ovarian cancer into molecular subtypes with different immune features. Some subtypes show enrichment of IFN-γ signaling, antigen presentation, cytotoxic lymphocyte markers, dendritic cells, and NK-cell activity, whereas others appear more immune-excluded (115). Risk scores based on cuproptosis-related genes have been associated with immune infiltration, prognosis, chemotherapy sensitivity, and potential immunotherapy response (115–118). These associations are hypothesis-generating and should not be interpreted as direct evidence that cuproptosis is already a clinically actionable biomarker. Functional studies are needed to determine whether manipulating copper-dependent death can causally remodel the ovarian tumor immune microenvironment.

### 3.7 Disulfidptosis and ovarian cancer

Disulfidptosis is a newly described death modality, and its direct evidence base in ovarian cancer is still limited. Mechanistically, it may be relevant because ovarian cancer cells often depend on SLC7A11-mediated cystine uptake and glutathione synthesis to manage oxidative stress. Under glucose deprivation, reduced NADPH production through the pentose phosphate pathway can impair thiol reduction, increase the GSSG/GSH ratio, and promote disulfide bond accumulation (119). This stress may disrupt actin dynamics through the Rac1-WAVE regulatory complex-Arp2/3 axis and associated cytoskeletal proteins. Current ovarian cancer-related evidence is mainly derived from pathway extrapolation and transcriptomic association, including links to redox status, metabolic stress, and candidate disulfidptosis-related genes (120). Therefore, this section should be interpreted as an emerging area rather than a mature therapeutic framework.

### 3.8 Autophagy-dependent cell death and ovarian cancer

Autophagy-dependent cell death (ADCD) is relevant to ovarian cancer because autophagy can either support tumor cell survival or contribute to cell death under specific stresses. Core autophagy regulators, including ATG3, ATG5, ATG7, ATG12, Beclin-1, LC3-II, and p62, have been reported to show altered expression in ovarian cancer and may vary according to histological subtype, stage, and treatment status (120). ATG5 and ATG7 are essential for autophagosome formation and may influence the crosstalk between autophagy and apoptosis (121). Beclin-1 activity is regulated partly through interaction with BCL-2, while LC3-II and p62 are commonly used to assess autophagic flux (122–124). In ovarian cancer models, modulation of these regulators has been associated with chemotherapy response, cell survival, and autophagy-associated death, but interpretation requires careful distinction between cytoprotective autophagy and true ADCD (124–126).

The role of ADCD in ovarian cancer is context dependent. Basal autophagy can promote survival under hypoxia, nutrient deprivation, and therapeutic stress, whereas excessive or dysregulated autophagic flux may degrade essential organelles and proteins and contribute to cell death (127). Pharmacological inhibition by agents such as 3-methyladenine can help test autophagy dependence in experimental systems, but these tools have limitations and off-target effects. ADCD may also influence migration, invasion, stemness, and peritoneal dissemination through effects on cytoskeletal regulation and metabolic adaptation (128–130). At present, ADCD should therefore be discussed as a biologically plausible but difficult-to-define death mechanism whose clinical utility depends on better assays for autophagic flux and pathway-specific causality.

## 4 Problems existing in current research

Although research on regulated cell death has expanded rapidly, several barriers still limit its clinical application in ovarian cancer. The most important problems include pathway crosstalk, uneven evidence depth across death modalities, resistance mechanisms, insufficient biomarkers, and limitations of available models and detection technologies.

### 4.1 The complexity and heterogeneity of the cell death network

Cell death pathways rarely operate independently in ovarian cancer. Apoptosis, ferroptosis, pyroptosis, cuproptosis, disulfidptosis, and autophagy interact through shared regulators, metabolic substrates, and stress responses. For example, SLC7A11 can support glutathione synthesis and suppress ferroptosis, but under glucose starvation it may also increase NADPH consumption and facilitate disulfide stress. Similarly, mitochondrial dysfunction, ROS accumulation, and lipid peroxidation may influence multiple death modalities at the same time. This crosstalk makes it difficult to attribute therapeutic effects to a single pathway and argues for experimental designs that directly test pathway specificity.

An additional layer of complexity is the interaction between metal ion-dependent death pathways and the immune microenvironment. Ferroptosis and cuproptosis are shaped by iron and copper homeostasis, respectively, and metal ion dysregulation may influence not only tumor cell vulnerability but also immune-cell function. Evidence from other tumor contexts suggests that metal ions can contribute to immune escape by regulating oxidative stress, angiogenesis, macrophage polarization, antigen presentation, and lymphocyte activity (102). In ovarian cancer, this metal ion–immune interface remains insufficiently validated and should be considered an important direction for future functional studies.

### 4.2 Limitations in the study of novel cell death mechanisms

Evidence depth is uneven across death modalities. Apoptosis and ferroptosis have relatively mature mechanistic and therapeutic literature in ovarian cancer, whereas cuproptosis and disulfidptosis remain much more exploratory. Ferroptosis research has focused heavily on the system Xc--GSH-GPX4 axis, but parallel systems such as FSP1-CoQ10, DHODH, and GCH1-BH4 require more ovarian cancer-specific work. Cuproptosis studies are promising but largely based on copper-related gene expression, mitochondrial metabolism, and bioinformatic prognostic models. Disulfidptosis requires especially cautious interpretation because direct functional validation in ovarian cancer is still limited.

### 4.3 The resistance mechanism of complexity and difficulties

Drug resistance is closely linked to defective or rewired cell death execution. Apoptosis impairment, enhanced antioxidant defense, altered iron or copper handling, autophagy-mediated stress tolerance, and drug efflux can all reduce the effectiveness of platinum and taxane therapy. Composite indices such as the CDI may be useful as conceptual tools for integrating multiple death-related features, but they should not be described as validated clinical predictors unless supported by independent cohorts and prospective evidence. Future work should define which components of these signatures are mechanistically causal and which are only correlative.

Targeted therapy challenges: Strategies directed at a single death pathway may be limited by biological redundancy, compensatory signaling, and tumor heterogeneity. For example, ferroptosis-inducing approaches can be counteracted by activation of antioxidant programs such as KEAP1-NRF2 signaling, increased expression of GPX4, FSP1, SLC7A11, or glutathione biosynthetic enzymes, and metabolic adaptation under therapeutic stress. Similarly, therapeutic manipulation of copper-dependent death may be complicated by systemic copper homeostasis, potential toxicity in normal tissues, and tumor cell adaptation through altered copper transport or reduced dependence on FDX1-related mitochondrial metabolism. These observations suggest that death pathway-targeted therapy is unlikely to be effective as a uniform strategy across all ovarian cancers. Rational combinations, such as co-targeting ferroptosis defense mechanisms, pairing death pathway modulation with platinum or PARP inhibitor treatment, or integrating metabolic inhibition with immune-based therapy, should therefore be evaluated in ovarian cancer-specific models with careful assessment of dose, sequence, toxicity, and predictive biomarkers. At present, such combinations should be considered preclinical or early translational strategies rather than established therapeutic regimens.

### 4.4 Lack of robust predictive biomarkers

Clinical translation also remains limited by the lack of robust predictive biomarkers. Single markers such as PD-L1, tumor mutational burden, or isolated death-related genes are unlikely to capture whether a tumor is vulnerable to apoptosis, ferroptosis, cuproptosis, disulfidptosis, or ADCD. A multi-parameter Death Vulnerability Signature (DVS) could in principle integrate transcriptomic, proteomic, metabolic, and spatial features, but such signatures remain hypothesis-generating. They should not be interpreted as validated tools for patient stratification or treatment selection at this stage. Before DVS-like models can be used clinically, they require analytical standardization, reproducible measurement platforms, predefined thresholds, independent external validation, prospective testing, and evidence that biomarker-guided intervention improves patient outcomes. In this context, artificial intelligence knowledge graphs may provide a useful methodological framework for developing interpretable multi-parametric biomarker systems. By linking molecular alterations, regulated cell death pathways, therapeutic agents, immune microenvironment features, clinical phenotypes, and treatment outcomes as structured entities and relationships, knowledge graph-based systems could help transform exploratory concepts such as CDI and DVS into testable models. Recent graph-based RAG-enhanced knowledge graph frameworks and broader healthcare knowledge graph studies support the feasibility of integrating heterogeneous biomedical information for explainable decision support. However, these approaches should be regarded as methodological references rather than direct evidence for ovarian cancer management, and ovarian cancer-specific validation would still be required before clinical application (131,132). Combination therapy is another challenge: multi-agent strategies may be rational, but their sequence, dose, toxicity, pharmacodynamic effects, and patient-selection criteria need to be defined in ovarian cancer-specific preclinical and clinical studies.

### 4.5 Limitations of research models and methodologies

Current models and methods also constrain the field. Two-dimensional cell cultures are useful for mechanistic screening but do not capture oxygen gradients, extracellular matrix interactions, immune components, or peritoneal dissemination. Xenografts provide *in vivo* information but may not reproduce human stroma, immune checkpoints, or histologic heterogeneity. Patient-derived organoids and *ex vivo* models preserve more tumor-specific features, but they remain expensive, technically variable, and incompletely standardized. Single-cell and spatial technologies can map cellular states and microenvironmental organization, but analytical pipelines and validation standards differ between studies. In addition, assays for lipid peroxidation, copper localization, disulfide stress, and autophagic flux require better spatial resolution, quantitative reliability, and pathway specificity.

## 5 Future research directions

Future research should move from broad pathway description toward focused, testable, and clinically anchored questions. Priority areas include integrated mapping of death pathway activity, biomarker validation, improved delivery systems, rational combinations, and dynamic monitoring of pathway engagement.

The metal ion–immune interface should be prioritized in future studies. Ferroptosis-related iron metabolism, cuproptosis-related copper handling, and pyroptosis-related inflammatory signaling may converge within the ovarian tumor microenvironment. Future work should examine whether iron-loaded macrophages, altered ferritin or ferroportin expression, copper transporter activity, or copper efflux programs affect tumor antigen release, dendritic cell cross-priming, cytotoxic lymphocyte infiltration, macrophage polarization, and immune checkpoint expression. Single-cell and spatial transcriptomic frameworks developed in other disease contexts may provide transferable strategies for resolving cell-state heterogeneity, metabolic–immune interactions, spatial immune niches, and death pathway activity within ovarian cancer tissues (33,34). Pan-cancer tumor microenvironment analyses may also provide comparative frameworks for distinguishing ovarian cancer-specific death–immune interactions from broader stromal, EMT-related, or immune-exclusion programs (68).

First, multi-omic studies should integrate genomic, transcriptomic, proteomic, spatial, and metabolic data from the same ovarian cancer specimens to define functional death states rather than relying only on gene expression. Candidate nodes such as KEAP1-NRF2-FSP1, YY1-FDX1-DLAT, and ATG5-Beclin-1-p62 should be evaluated across tumor cells and stromal or immune compartments. Second, biomarker-guided trials should be designed cautiously, using validated assays and predefined thresholds rather than exploratory scores alone. Third, delivery systems such as targeted liposomes, polymeric micelles, or mitochondria-directed nanoparticles may improve therapeutic windows, but they require rigorous toxicity and pharmacodynamic assessment. Fourth, combination regimens should be optimized for mechanism, timing, and patient selection rather than presented as uniformly synergistic. AI-assisted and network-based approaches may help address this challenge by decomposing multi-agent effects into pathway-specific and cell-state-specific contributions. For example, graph-based models, causal network inference, and multi-omic integration frameworks could be used to map how candidate agents affect ferroptosis defense, apoptotic priming, copper metabolism, autophagic flux, immune activation, and compensatory survival pathways. These computational approaches should not replace experimental validation, but they may help prioritize rational drug combinations, define sequencing strategies, and identify biomarkers for ovarian cancer-specific preclinical testing (64). Finally, imaging and liquid-biopsy approaches may help monitor pathway engagement, including copper uptake, lipid peroxidation, redox imbalance, and death effector gene alterations, but these tools need technical validation before routine clinical use.

## 6 Conclusion

Regulated cell death offers a useful framework for understanding ovarian cancer progression, treatment resistance, and tumor–immune interactions. Among the pathways reviewed, apoptosis and ferroptosis currently have the strongest ovarian cancer-specific evidence, particularly in relation to chemotherapy response, oxidative stress, and drug resistance. Necroptosis, pyroptosis, cuproptosis, disulfidptosis, and autophagy-dependent cell death are also biologically relevant, but their evidence base is more variable and often remains preclinical or transcriptomic.

The main translational value of these pathways lies in explaining heterogeneous treatment responses and identifying testable vulnerabilities rather than providing immediately applicable clinical tools. In particular, metal ion-dependent pathways may connect metabolic stress with immune regulation, while multi-parametric models may eventually support refined patient stratification. However, concepts such as the Cell Death Index, Death Vulnerability Signature, and death interactome mapping should currently be regarded as hypothesis-generating frameworks rather than validated decision-making tools.

Future studies should prioritize ovarian cancer-specific functional validation, standardized biomarker assays, patient-derived and spatially resolved models, independent clinical cohorts, and biomarker-guided trials. Only through this evidence-building process can regulated cell death pathways be translated from mechanistic concepts into clinically meaningful strategies for ovarian cancer.
